# Advanced Clustering for Mobile Network Optimization: A Systematic Literature Review

**DOI:** 10.3390/s25237370

**Published:** 2025-12-04

**Authors:** Claude Mukatshung Nawej, Pius Adewale Owolawi, Tom Mmbasu Walingo

**Affiliations:** 1 Department of Electrical, Electronics, and Computer Engineering, University of Kwa-Zulu Natal, Durban 4041, South Africa; walingo@ukzn.ac.za; 2 Department of Computer Systems Engineering, Tshwane University of Technology, Pretoria 0183, South Africa; owolawipa@tut.ac.za

**Keywords:** advanced clustering techniques, mobile network optimization, quality-of-service (QoS) prediction

## Abstract

5G technology represents a transformative shift in mobile communications, delivering improved ultra-low latency, data throughput, and the capacity to support huge device connectivity, surpassing the capabilities of LTE systems. As global telecommunication operators shift toward widespread 5G implementation, ensuring optimal network performance and intelligent resource management has become increasingly obvious. To address these challenges, this study explored the role of advanced clustering methods in optimizing cellular networks under heterogeneous and dynamic conditions. A systematic literature review (SLR) was conducted by analyzing 40 peer-reviewed and non-peer-reviewed studies selected from an initial collection of 500 papers retrieved from the Semantic Scholar Open Research Corpus. This review examines a diversity of clustering approaches, including spectral clustering with Bayesian non-parametric models and K-means, density-based clustering such as DBSCAN, and deep representation-based methods like Differential Evolution Memetic Clustering (DEMC) and Domain Adaptive Neighborhood Clustering via Entropy Optimization (DANCE). Key performance outcomes reported across studies include anomaly detection accuracy of up to 98.8%, delivery rate improvements of up to 89.4%, and handover prediction accuracy improvements of approximately 43%, particularly when clustering techniques are combined with machine learning models. In addition to summarizing their effectiveness, this review highlights methodological trends in clustering parameters, mechanisms, experimental setups, and quality metrics. The findings suggest that advanced clustering models play a crucial role in intelligent spectrum sensing, adaptive mobility management, and efficient resource allocation, thereby contributing meaningfully to the development of intelligent 5G/6G mobile network infrastructures.

## 1. Introduction

### 1.1. Background

The convergence of artificial intelligence (AI) and data-centric technologies has become pivotal for next-generation communication systems, enabling intelligent and adaptive network optimization. Modern mobile networks must dynamically interpret complex traffic patterns, respond to user mobility, and adapt to evolving environmental conditions to ensure high performance and reliability, particularly in heterogeneous and densely deployed infrastructures. To meet these demands and enhance users’ quality of experience (QoE), advanced clustering techniques have emerged as essential tools for mobile network optimization.

In this study, clustering refers explicitly to unsupervised machine learning techniques used for analysing and managing mobile network data. It excludes the physical grouping of network nodes, such as base stations or data centres, and instead focuses on algorithmic approaches such as spectral clustering (e.g., Bayesian non-parametric models), density-based clustering (e.g., DBSCAN), and deep representation-based methods (e.g., DEMC and DANCE). These techniques enable feature discovery, anomaly detection, and dynamic quality-of-service (QoS) management within intelligent mobile infrastructures.

The ongoing global deployment of fifth generation (5G) networks and the anticipated evolution toward sixth generation (6G) networks introduce new dimensions of complexity in mobile communications. 5G supports enhanced mobile broadband (eMBB), massive machine-type communications (mMTC), and ultra-reliable low-latency communications (URLLC), collectively enabling services and automation at unprecedented scales. By contrast, 6G is envisioned to push beyond these capabilities, targeting terabit-per-second peak data rates, sub-millisecond end-to-end latencies, and native integration of sensing and communication. The 6G vision emphasizes the use of the upper mid-band spectrum (7–20 GHz) for enhanced capacity, open radio access networks (O-RANs) for architectural flexibility, and AI-native designs for self-optimizing network behaviour [1,2].

While integrated sensing and communications (ISAC) and dual-functional radar-communication (DFRC) are related concepts, they are not synonymous. ISAC represents a broader design paradigm in which communication and sensing functionalities are jointly integrated within a unified wireless infrastructure. In ISAC, spectrum, waveforms, and hardware are shared to support both data transmission and environmental perception, making it a key enabler of 6G’s intelligent, perceptive networking capabilities.

In contrast, DFRC refers to a specific system-level realization of this concept, typically at the physical layer, where a transceiver performs both radar sensing and data communication simultaneously using shared or co-designed waveforms. DFRC research focuses on waveform optimization, precoding, and beamforming to achieve dual functionality with minimal performance trade-offs. Thus, DFRC can be viewed as a subset or implementation approach within the broader ISAC framework.

Recent work on hybrid radar fusion demonstrates how monostatic DFRC base stations can fuse uplink and downlink sensing measurements to improve range and angle estimation accuracy [3]. Further, waveform and precoder design for DFRC systems has been extensively studied [4], while secure full-duplex ISAC architectures have recently been explored to enhance confidentiality and interference mitigation [5]. Together, these developments underscore the importance of integrating ISAC (as a system-level framework) and DFRC (as a physical-layer realization) into future discussions on spectrum-aware clustering and sensing-aware resource allocation in next-generation networks [6].

Moreover, the International Telecommunication Union (ITU) has identified six key pillars defining the 6G framework: (i) massive communications, (ii) ubiquitous connectivity, (iii) hyper-reliable and low-latency communication, (iv) AI and communication, (v) immersive communication, and (vi) integrated sensing and communication [7]. These pillars guide the evolution of intelligent, flexible, and perceptive communication networks that align with future digital transformation objectives.

As these networks grow more complex, optimization objectives extend beyond traditional load balancing and signal strength enhancement. Current research increasingly focuses on dynamic resource allocation, fault detection, traffic classification, anomaly detection, intelligent handovers, and service orchestration. To address these challenges, clustering algorithms such as DBSCAN, K-means, Bayesian non-parametric models, and deep representation-based methods like DEMC and DANCE have gained widespread adoption. Table 1 summarizes the main clustering algorithms, outlining their fundamental principles, objectives, and mathematical formulations used in mobile network optimization.

Coupled with advances in machine learning, clustering enables autonomous network adaptation with minimal human intervention, thereby improving operational efficiency and resilience under dynamic conditions.

The economic implications of these advances are significant. For example, the World Economic Forum estimates that 5G alone could contribute more than USD 13 trillion to the global economy by 2030 and USD 13.2 trillion by 2035 [8]. The mobile network analytics market, valued at USD 6.53 billion in 2024, is projected to reach USD 18.97 billion due to automation and optimization priorities [9]. Empirical studies have shown that clustering integrated with machine learning can achieve up to 98.8% accuracy in anomaly detection [10], 89.4% improvement in data delivery [11], and a 43% enhancement in handover prediction [12], demonstrating the practical value of clustering-based methods in operational networks. It is important to note that these results were obtained from existing peer-reviewed literature and did not originate from the original experimental work in this review. They demonstrated the effectiveness of advanced clustering methods when integrated with machine learning models.

Moreover, it should be noted that a distinction must be made between narrative reviews and systematic literature reviews (SLRs). Narrative reviews provide broad, qualitative insights but often lack transparency and reproducibility, leading to selective reporting. In contrast, SLRs follow a rigorous and reproducible approach for identifying, selecting, and analyzing relevant studies. This study followed the Preferred Reporting Items for Systematic Reviews and Meta-Analyses (PRISMA) methodology [13,14] to ensure objectivity and reliability through well-defined search strategies, selection criteria, and data extraction processes.

### 1.2. Research Gaps

Despite substantial contributions to cellular network optimization, most existing literature reviews either adopt a narrow technical focus or follow a narrative approach that lack methodological rigor. Specifically, there is no unified framework that systematically compares clustering techniques across different network generations, traffic types, or deployment scenarios. Algorithm-specific parameters, performance metrics, and real-world evaluation environments have rarely been reported in an integrated manner, limiting the ability of researchers to benchmark approaches, evaluate cross-context applicability, and design generalizable clustering solutions. Several existing surveys, such as those on clustering in wireless sensor networks and anomaly detection in 5G, have provided foundational insights. However, they often focus on narrow domains or fail to comprehensively explore deep-representation-based techniques. Unlike prior studies, this review offers a unified analysis across heterogeneous 5G/6G environments, integrates deep learning and federated learning clustering models, and assesses their utility in adaptive QoS management, filling a key research gap.

Furthermore, evaluating clustering algorithms across both 4G and 5G contexts offers unique insights into their scalability and adaptability. With 5G introducing architectural innovations such as network slicing, edge orchestration, and AI-native management, and 6G emphasizing integrated sensing, pervasive intelligence, and immersive services, cross-generational and forward-looking analyses are essential for identifying algorithmic strengths and designing robust solutions aligned with 6G vision.

### 1.3. Objectives

This study conducts a systematic review of advanced clustering techniques, including deep representation-based, spectrum-based, and density-based approaches, for feature discovery, anomaly detection, and adaptive quality of service (QoS) estimation in heterogeneous and dynamic mobile network environments.

The major contributions of this study are as follows:

(i.) Classification of clustering methods and their applications in mobile networking;
(ii.) Review of algorithmic parameters and performance metrics;
(iii.) Exploration of integration with deep learning, edge computing, and federated learning;
(iv.) Identification of open challenges and research direction; and
(v.) Consolidated reference for practitioners and researchers exploring clustering for adaptive QoS in 5G/6G mobile networks

The rest of this paper is organized as follows. Section 2 presents the research methodology. Section 3 provides the results and thematic analysis. Section 4 presents the conclusion and future research directions.

## 2. Research Methodology

For clarity, the clustering techniques covered in this review are grouped into three primary categories: (i) spectrum-based clustering, including Bayesian and K-means methods; (ii) density-based clustering such as DBSCAN; and (iii) deep representation-based clustering including DEMC and DANCE. This study adopts the SPICE framework (Setting, Perspective, Intervention, Comparison, Evaluation) to structure the research question and guide the methodology.

### 2.1. Research Question and Framework

The main research questions guiding this systematic review are: “How can advanced clustering techniques, including spectral clustering, density-based, and deep representation-based clustering, be applied to enhance feature discovery, anomaly detection, and adaptive quality-of-service (QoS) prediction in heterogeneous and dynamic mobile network environments?”

To ensure a structured, comprehensive, and contextually relevant study of the challenges in intelligent mobile network management, the SPICE framework was employed to formulate this research question. SPICE is widely used to structure focused and application-oriented research questions suitable for practical deployment.

In this study, the setting was defined as a mixed and dynamic cellular network environment, capturing the non-static and heterogeneous nature of modern mobile networks. The Perspective focused on researchers and system designers, ensuring that the question reflected stakeholder priorities and real-world concerns. The interventions included spectral clustering, density-based methods, and deep representation-based clustering, highlighting the core analytical strategies under investigation. Although a direct comparison was not incorporated, given the emphasis on innovation rather than benchmarking, the framework remained effective in supporting methodological advancement. Finally, the Evaluation emphasized measurable outcomes such as feature discovery, anomaly detection, and adaptive QoS prediction, aligning the research objectives with performance-driven and practically relevant goals. These elements of the SPICE framework are summarized in Table 2, which outlines the Setting, Perspective, Intervention, Comparison, and Evaluation components used to guide this systematic review.

The main research question developed for this study is supported by three sub-research questions designed to provide a structured pathway for detailed analysis.

First, it seeks to identify the main categories of clustering techniques and examine how each category aligns with specific mobile network use cases. This includes evaluating the suitability of spectrum-based, density-based, and deep representation-based clustering methods for key tasks such as anomaly detection, feature discovery, and adaptive QoS management.

Second, the review explores how algorithmic parameters and evaluation metrics influence clustering performance. By analyzing the impact of parameter choices (e.g., cluster size and, distance metrics) and performance indicators (e.g., accuracy, latency, and throughput), this study aims to highlight the critical design factors that affect the reliability and efficiency of clustering outcomes.

Third, the review investigates how clustering approaches are integrated with advanced technologies, including deep learning, and machine learning. The objective is to understand how these integrations enable the intelligent, real-time, and scalable optimization of mobile networks in increasingly complex and heterogeneous environments.

Collectively, these sub-questions support the main research aim and help to uncover emerging trends and evolving practices in the field. The sub-research questions were as follows:

1. What are the major categories of clustering techniques and how do they align with mobile network use cases?
2. How do algorithmic parameters and evaluation metrics influence clustering performance?
3. In what ways are clustering approaches integrated with deep learning, edge computing, and machine learning to support intelligent, real-time, and scalable network optimization?

### 2.2. Review Protocol

This review fundamentally emphasizes the way in which these techniques have been used to tackle the key challenges of feature discovery, anomaly detection, and adaptive quality-of-service (QoS) prediction in dynamic and heterogeneous mobile network environments. The objective is to synthesize the existing body of knowledge and pinpoint evolving trends, performance outcomes, and methodological patterns that can inform future research and engineering practices in mobile network optimization.

#### 2.2.1. Search Strategy and Eligibility Criteria

The search strategy used multiple databases (IEEE Xplore, Scopus, Web of Science, SpringerLink), with the following keywords: “clustering,” “QoS prediction,” “5G,” “6G,” “adaptive networks,” “machine learning,” “unsupervised learning,” and “heterogeneous mobile networks.” The inclusion criteria were as follows: (i) peer-reviewed, (ii) English, (iii) published between 2000 and 2024, with priority on recent 5G/6G papers; and (iv) direct relevance to clustering in mobile networks. Dissertations, patents, non-peer-reviewed materials, or studies lacking empirical evaluation were excluded. After screening 10,000+ entries, 500 were retained, and 40 met all high-quality inclusion criteria.

#### 2.2.2. Study Selection Process

**Figure 1:**
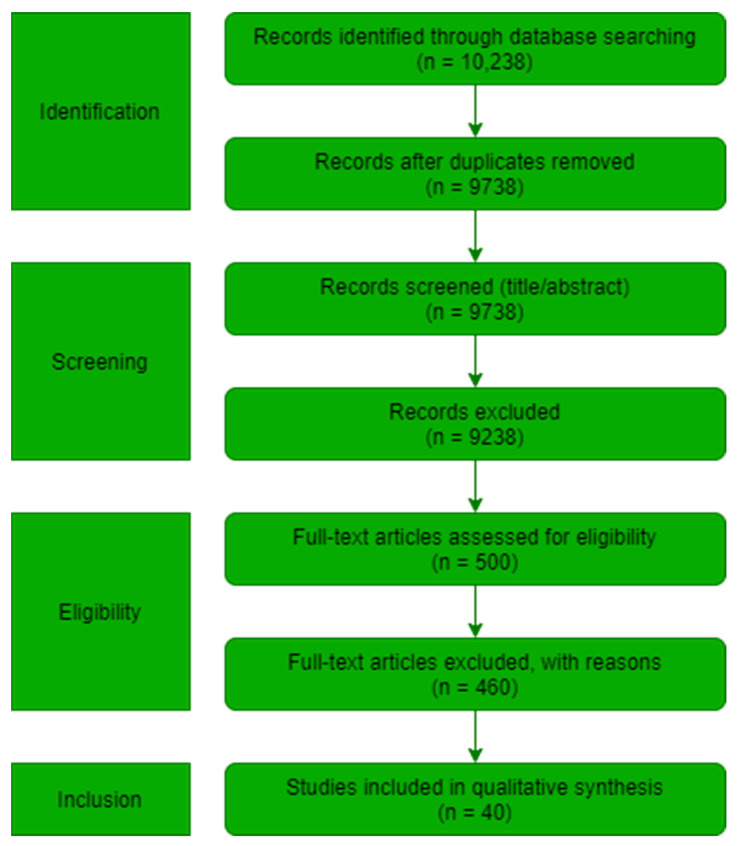
PRISMA flow diagram outlining the systematic review protocol and decision flow.

The remaining 40 studies were subjected to full-text assessment, and all were deemed eligible and retained for the final synthesis. The study identification, screening, eligibility, and inclusion phases were visually documented using the PRISMA 2020 [13,14] flow diagram reported in Figure 1.

To contextualize these publication trends, Table 3 summarizes the PRISMA stages and corresponding study counts, illustrating the identification, screening, eligibility, and inclusion processes that led to the final set of 40 studies included in this review.

**Figure 2:**
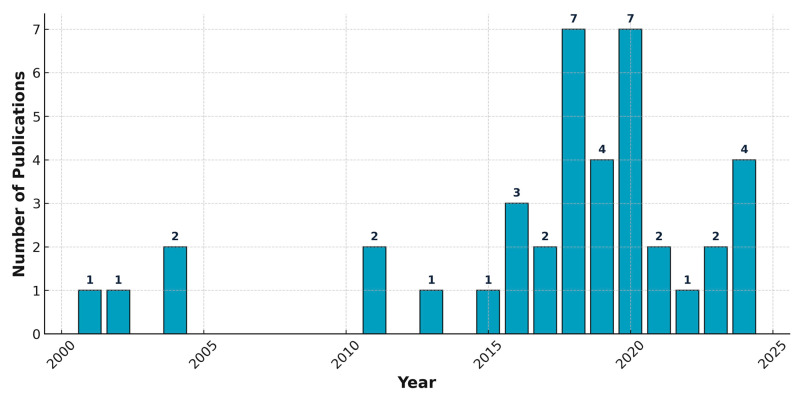
Annual distribution of peer-reviewed journal and conference publications in English related to clustering in mobile networks, covering the period from 2000 to 2024.

A notable increase in research output was observed from 2017 onwards, with peak publications recorded in 2018 and 2020, suggesting heightened research interest and activity during this period. In contrast, earlier years (2000–2010) showed relatively sporadic and low publication rates, indicating limited early exploration of the topic. The post-2017 surge may reflect the impact of emerging technologies, methodological advancements, or the growing recognition of the importance of the research area. Following is Figure 2, which illustrates the annual distribution of peer-reviewed journal and conference publications in English related to clustering in mobile networks, spanning the period from 2000 to 2024.

#### 2.2.3. Data Extraction and Management

Table 4 summarizes the reasons for excluding studies during the full-text eligibility assessment phase. Each paper was evaluated against the predefined inclusion and exclusion criteria to ensure methodological rigor and thematic relevance. The exclusion counts reflect the dominant reasons why studies did not meet the review’s eligibility requirements, supporting the transparency and reproducibility of the selection process.

As summarized in Table 4, a total of 460 studies were excluded during the full-text review phase. The most common exclusion reason (*n* = 210) was that the study did not focus on clustering within mobile network environments, often addressing unrelated application domains such as IoT, healthcare, or social media. A further 120 papers were excluded for lacking empirical or quantitative evaluation, while 85 studies fell outside the defined publication period (2000–2024) or were otherwise out of scope. Additionally, 30 publications were removed due to non-peer-reviewed status or inaccessibility, and 15 entries were excluded as duplicates or due to data inconsistencies.

#### 2.2.4. Risk of Bias and Quality Assessment

Quality assessment of the included studies was performed using the AMSTAR (A Measurement Tool to Assess Systematic Reviews) checklist. The evaluation focused on design rigor, empirical validation, and reproducibility of each study. The results of the appraisal process are summarized in Table 5, which includes assessments of the 11 AMSTAR criteria.

Why AMSTAR? AMSTAR provides a concise, well-known checklist for assessing methodological transparency, search comprehensiveness, and reporting quality of systematic reviews. Items such as whether priori criteria were specified, whether a comprehensive search was performed, and whether study quality was considered in conclusions, are broadly applicable beyond health sciences and help to establish reproducible review practice. We used AMSTAR for engineering studies as a framework to assess reporting and transparency rather than as a prescriptive medical instrument. Where AMSTAR items were not directly applicable to algorithmic/empirical engineering work (for example, items focused on patient outcomes), we:

(a) explicitly noted non-applicability and documented the reason, and
(b) replaced or supplemented those items with engineering-relevant checks (e.g., whether datasets, code, or experimental settings were reported, whether performance metrics and computational costs were provided).

Table 5 presents the AMSTAR quality appraisal summary.

#### 2.2.5. Data Synthesis

Owing to methodological and application heterogeneity, a narrative synthesis approach was adopted instead of a meta-analysis. The findings were thematically grouped based on the type of clustering technique and associated application area (e.g., feature discovery or anomaly detection). This allowed for a detailed interpretation of the literature while preserving methodological distinctions.

#### 2.2.6. Protocol Registration

To ensure transparency and reproducibility, the full protocol for this systematic literature review was registered in the Open Science Framework (OSF).

## 3. Results and Thematic Analyses

In this section, the findings of the systematic literature review, combining both quantitative and qualitative intuitions, provide a comprehensive understanding of how clustering techniques are applied in mobile network optimization. The first part of the section emphasizes quantitative results, combining the publications per year, distribution of network environments, clustering techniques, and application domains. Following this, thematic analysis identifies and interprets recurring patterns and conceptual trends within the studies, shedding light on the motivations, challenges, and innovations that shape the current research landscape.

### 3.1. Quantitative Results

Table 6 presents the characteristics of the included studies.

#### 3.1.1. Network Environment Studied

The selected studies have employed a wide range of network environments. 5G networks were the most frequently studied, appearing in 10 studies, followed by general mobile or cellular networks in 9 studies. Ad hoc environments, including Mobile Ad Hoc Networks (MANETs) and general ad hoc setups, were considered in 5 studies. LTE/4G networks were featured in three studies, whereas IoT and vehicular ad-hoc networks (VANETs) were the focus of two studies each. Several specialized environments, including cognitive radio networks, vehicular cloud networks, and mobile edge computing, have appeared in only one study. Remarkably, one study did not specify a network environment.

The reviewed studies were conducted in a wide range of network environments, showing a growing pattern of mobile and wireless communication systems. 5G networks have evolved as the most frequently studied environment, presenting 10 of the 40 studies, underlining their relevance to modern mobile network optimization challenges. While foundational clustering methods from earlier decades were included for historical completeness, the majority of the selected studies (60%) were from post-2018, reflecting current trends in 5G/6G network research. Older studies are briefly summarized to contextualize the evolution of clustering approaches in mobile networks.

**Figure 3:**
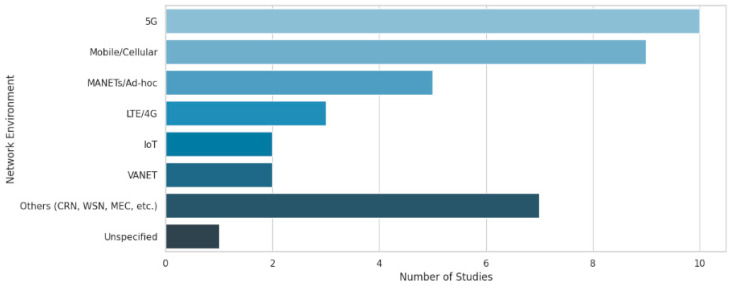
Distribution of network environments in selected studies.

The bar chart in Figure 3 shows the number of studies per network environment category.

#### 3.1.2. Clustering Techniques Used

**Figure 4:**
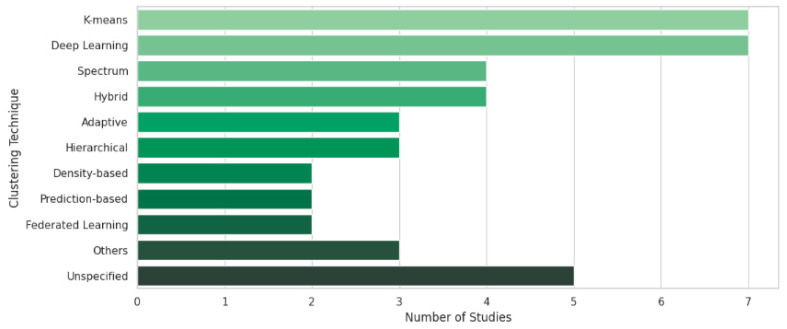
Frequency of clustering techniques employed.

This review reveals diverse clustering approaches. Both K-means clustering and deep learning/representation-based methods were the most common methods used in seven studies. These were followed by spectral clustering and hybrid techniques, each of which was found in four studies. Adaptive and hierarchical clustering approaches were applied in three studies. Techniques such as density-based, prediction-based, and federated learning appeared in two studies. Other specialized approaches (e.g., ensemble, time-aware, dynamic, and geographical clustering) were found in one to two studies. Five studies did not specify the clustering technique. Figure 4 shows the clustering methods with the corresponding study counts.

Several performance metrics critical to clustering in 5G/6G networks were identified, including the accuracy, precision, recall, F1-score, silhouette coefficient, spectral efficiency, energy efficiency, latency, and throughput. Spectral efficiency and latency are crucial for meeting the demands of next-generation networks. Future studies should therefore balance quantitative evaluation with qualitative insights, including interpretability, adaptability, and robustness, to achieve a more holistic understanding of clustering effectiveness in next-generation networks.

#### 3.1.3. Application Domains

**Figure 5:**
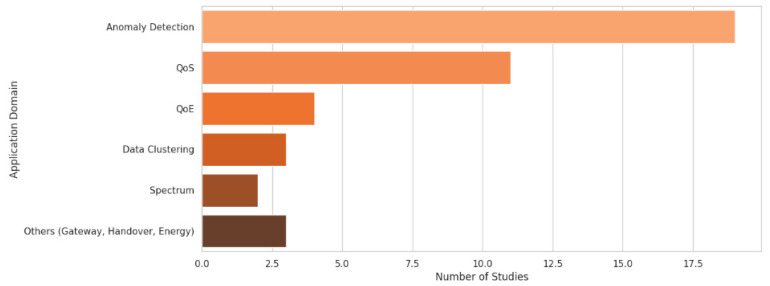
Application areas addressed by reviewed studies.

A substantial portion of the reviewed work focused on anomaly detection, appearing in 19 of the 40 studies, demonstrating its central role in mobile network security and performance monitoring. Quality of service (QoS)-related applications were the second most prevalent and addressed in 11 studies. Quality of Experience (QoE) appeared in four studies, indicating a moderate focus on user-centric optimization. Other applications such as data clustering/collection (three studies) and spectrum management (two studies) were less common. A small number of studies (one each) focused on niche areas such as gateway management, handover prediction, and energy efficiency. Figure 5 shows the number of studies per application.

It is important to note that this review does not aim to simulate specific 5G/6G scenarios but rather consolidates the literature on how clustering techniques support adaptive QoS in heterogeneous mobile environments. Therefore, detailed simulation parameters (e.g., network topologies) were beyond the scope of this study.

### 3.2. Thematic Analysis

Thematic analysis involves identifying, analyzing, and interpreting patterns within qualitative data. To complement the quantitative findings, a thematic analysis was conducted to uncover recurring conceptual patterns and emerging trends in the literature.

#### 3.2.1. Clustering Techniques and Their Applications

This section categorizes the clustering techniques based on their technical formulation, implementation approaches, and network specific applications as presented in Table 7.

- Implementation approaches

**Figure 6:**
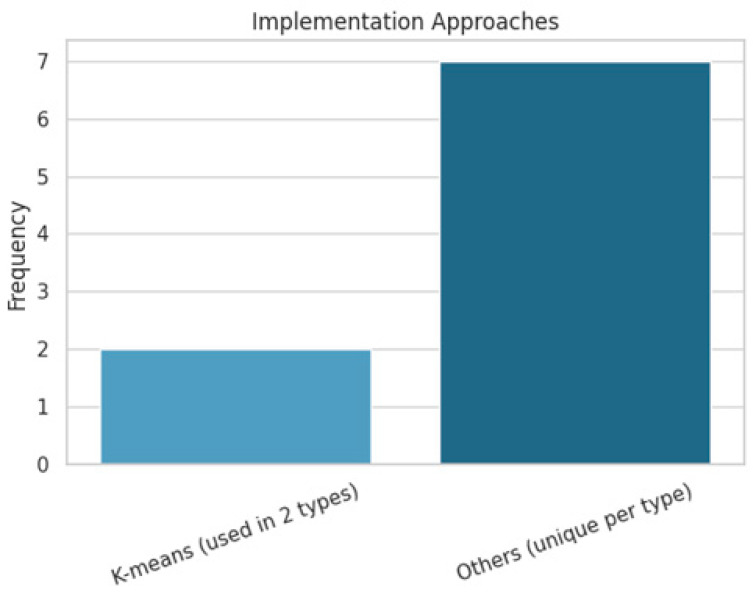
Distribution of implementation approaches adopted across different clustering methods, illustrating the relative reliance on common algorithms (e.g., K-means) versus technique-specific or uniquely tailored implementations.

Our review identified 14 distinct implementation approaches distributed across nine clustering techniques. Among these, K-means clustering emerged as the only technique mentioned across the two different clustering types, underscoring its popularity and adaptability in mobile network optimization scenarios. All other implementation methods were unique to their respective technique types, indicating a high degree of customization tailored to specific research contexts or performance goals. Figure 6 shows a visual representation of the implemented approaches.

- Performance metrics

**Figure 7:**
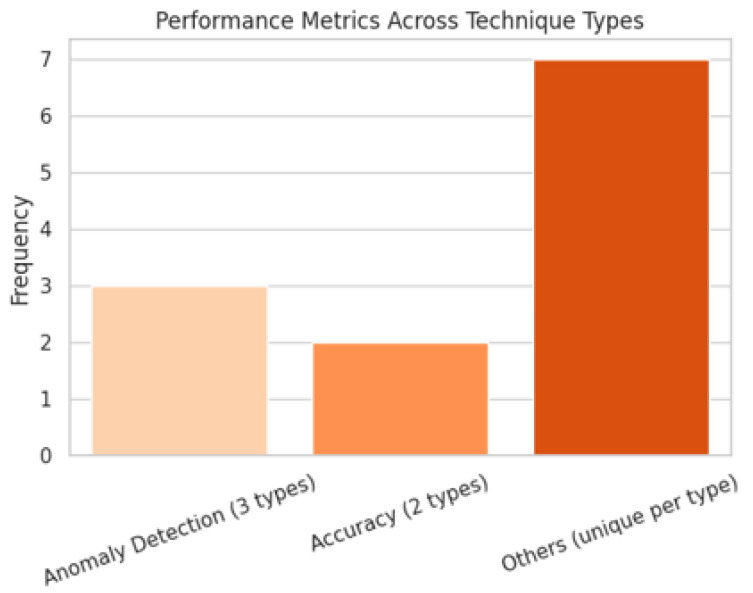
Frequency distribution of performance metrics across clustering technique types.

The studies employed 12 unique performance metrics to evaluate the clustering effectiveness. Notably, anomaly detection was the most prevalent, appearing in the three different techniques, reflecting its central role in securing and monitoring mobile networks. Accuracy was the second most common metric, mentioned for both techniques. The remaining metrics were associated with only one technique, highlighting a fragmented landscape where performance evaluation is highly contextual and often technique specific. Figure 7 shows a visualization of the performance metrics across techniques.

- Adaptation capabilities

**Figure 8:**
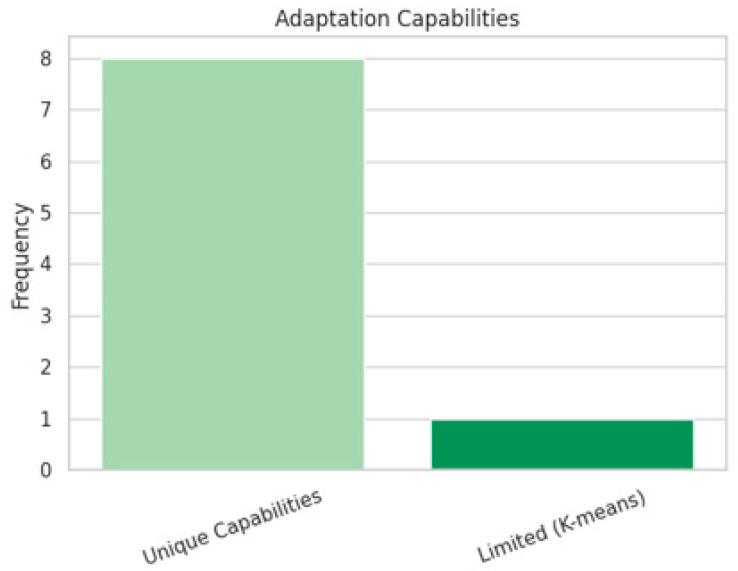
Adaptation capabilities of clustering methods, showing that most exhibit unique adaptability, while K-means remains limited.

We identified nine distinct adaptation capabilities across clustering techniques, each uniquely tied to their respective approaches. These capabilities range from adapting to channel conditions, traffic variability, and node mobility, to dynamically responding to environmental changes. Interestingly, K-means clustering was singled out for its limited adaptation capabilities, reinforcing its perception as a baseline technique that may lack the responsiveness required in highly dynamic environments. These differences in adaptability across clustering techniques are visually summarized in Figure 8, highlighting how most methods demonstrate unique adaptation strengths, whereas K-means remains notably limited.

#### 3.2.2. Network Performance Enhancement

This paragraph organizes clustering applications around ten major enhancement areas and evaluates their implementation methods, benefits, and limitations. Table 8 presents the network performance enhancement.

**Figure 9:**
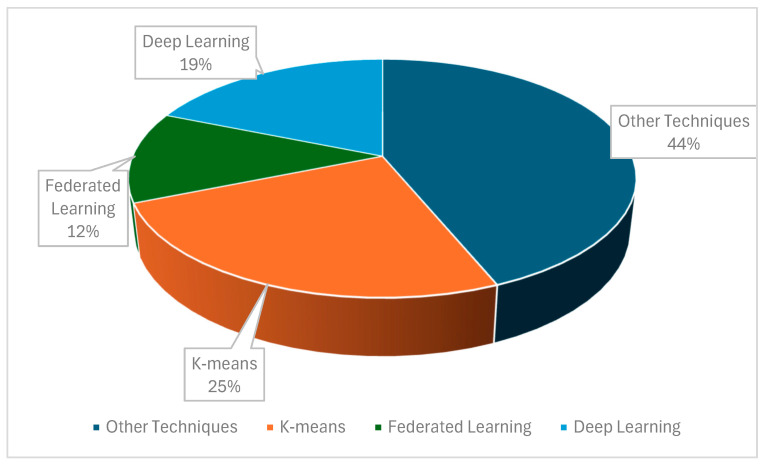
Distribution of clustering implementation methods categorized by performance evaluation domains, illustrating the extent to which different techniques are applied across distinct performance areas.

Sixteen distinct implementation methods were identified. Among these, certain techniques, most notably Deep Learning, Federated Learning, and K-means, have repeatedly appeared, demonstrating their versatility and adaptability across different domains of network optimization. These methods were not confined to single-purpose use; instead, they spanned multiple enhancement areas such as anomaly detection, QoS prediction, and handover management. Figure 9 provides the distribution of clustering implementation methods categorized by performance evaluation domains.

Recurring benefits were evident throughout the reviewed implementations. Many approaches have reported gains in prediction accuracy and Quality of Service (QoS), suggesting that clustering-based models are instrumental in addressing performance variability in mobile networks. However, the associated limitations were predominantly domain specific. This highlights a persistent contextual challenge: while clustering techniques show promise in enhancing network functionality, their practical deployment often requires customization to fit the unique constraints of each application area.

#### 3.2.3. Adaptive Mechanisms and Dynamic Response

Clustering techniques in mobile networks are increasingly incorporating adaptive mechanisms to address the dynamic nature of real-world environments. These mechanisms are essential for maintaining performance under conditions such as user mobility, fluctuating traffic patterns, and shifting spectrum availability. Several studies have demonstrated real-time adaptation capabilities. For instance, Ali et al. [15] and Gajic et al. [16] exhibited responsiveness to evolving network conditions by processing and adjusting streaming data in real time. Mobility-aware clustering is another critical adaptation domain. Techniques such as prediction-based clustering proposed by Sivavakeesar and Pavlou [17] and federated clustering for handover prediction explored by Nivitha et al. [18] focus on preserving service quality despite the continual movement of users or nodes across network boundaries. In the context of traffic-adaptive clustering, methods such as the self-adaptive deep learning model introduced by Fernández Maimó et al. [19] and the adaptive vehicular clustering network model by Kaleibar and St-Hilaire [20] adjust the clustering behavior in response to varying traffic loads and patterns, thereby enabling stable and efficient communication. Adaptation to spectrum dynamics was also explored. Notable contributions include adaptive cooperative sensing mechanisms by Pêrez and Santamaría [21] and time-variant spectral clustering approaches such as those developed by Sun et al. [22], both of which dynamically adjust spectrum usage strategies based on real-time measurements. QoS and QoE remain central to adaptive efforts. Yin et al. [23] applied fuzzy clustering for QoS prediction, whereas John and Thangaraj [24] implemented QoE-driven anomaly detection, demonstrating how adaptive clustering can align with user experience metrics in complex environments. From a resource management perspective, techniques such as adaptive DBSCAN combined with deep reinforcement learning by Elsayed and Erol-Kantarci [25] and dynamic DBSCAN-based methods by Ren and Xu [26] adapt to the availability and demand for computational or network resources.

Finally, the hybrid adaptive framework represents the convergence of the multiple adaptation strategies. These include reinforcement learning-enhanced clustering by Kim et al. [27] and co-clustering techniques combined with logistic regression by Kassan et al. [28], which aim to improve responsiveness while maintaining the overall system stability. However, despite these advances, several challenges remain. These include managing the computational complexity, balancing stability with responsiveness, ensuring scalability across large heterogeneous networks, and validating techniques in real-world scenarios. Together, these insights emphasize the growing sophistication and importance of adaptive mechanisms in clustering-based mobile network optimization.

#### 3.2.4. Integration Challenges and Solutions

Clustering techniques offer promising advancements in mobile network optimization; however, their integration into real-world systems remains fraught with technical and operational challenges. This theme synthesizes the key barriers to implementation and highlights innovative solutions proposed in the literature. One of the foremost obstacles is the heterogeneity of network environments. Seamless operations across various platforms, such as vehicular ad hoc networks (VANETs) and legacy mobile systems (UMTS), require significant adaptation. Benslimane et al. [29] and Xu et al. [30] illustrated approaches to unify VANET and WSN/IoT clustering within the broader 5G ecosystem.

Scalability and real-time performance are critical concerns, particularly for high-velocity data streams and dense user environments. Techniques such as NetWalk [15] and DBSCAN combined with LSTM-driven deep reinforcement learning [25] have demonstrated how adaptive algorithms can help maintain responsiveness and reliability at scale. Privacy and data security have become increasingly pertinent. Federated learning-based clustering methods, such as those proposed by Fernández Maimó et al. [19] and extended by Stenhammar et al. [31], preserve user privacy by decentralizing data processing while enabling efficient model training and cluster formation. Another significant challenge is the adaption to network changes. Real-time fluctuations in topology, user behavior, and service demand require clustering mechanisms that are responsive and robust. Incremental time-aware clustering [16] and dynamic clustering for vehicular cloud networks [20] offer effective methods for continuous adaptation to evolving scenarios. The issue of legacy integration, incorporating advanced clustering into established network infrastructures, has been addressed by Fernández Maimó et al. [21] through MEC-based anomaly detection, and by Ren and Xu [26] with DBSCAN adaptations for ultra-dense networks.

As clustering increasingly engages with multi-dimensional datasets, ensuring an accurate representation and low-noise inputs becomes vital. Approaches involving spectrum feature vector clustering [22] and denoising auto-encoders [23] have been employed to address these data-related complexities. The literature also reveals efforts to balance optimization trade-offs, particularly between the accuracy, latency, and energy consumption. Solutions include the QoE-driven anomaly detection frameworks by Murudkar and Gitlin [28] and energy-efficient VANET clustering techniques by Padmanabhan et al. [32]. Despite these advances, the interpretability of clustering decisions remains a challenge. Few studies have provided transparent frameworks for understanding or validating cluster outputs, which is a critical area for future research.

Finally, in efforts toward cross-layer optimization, studies such as those by Ali et al. [15] proposed multi-channel cognitive radio networks that integrate clustering across different OSI layers to improve overall network efficiency. Similarly, energy efficiency, particularly in resource-constrained environments such as WSNs, is addressed using energy-aware clustering approaches [30]. Researchers advocate the development of standardized benchmarking frameworks, deployment of hybrid and flexible clustering designs, broader integration into edge computing platforms, and incorporation of clustering into comprehensive AI/ML pipelines. These advancements should be validated through real-world pilot implementations to bridge the gap between theory and practice.

## 4. Conclusions

This systematic review highlights an evolving landscape in the application of clustering techniques for mobile network optimization. While traditional algorithms such as K-means and hierarchical clustering remain widely utilized, there has been a marked shift toward more sophisticated approaches, including adaptive, hybrid, and deep representation-based clustering. These methods are increasingly being adopted to address the multifaceted challenges posed by next-generation network environments, particularly within 5G, IoT, and vehicular networks, which are characterized by high user mobility, diverse service requirements, and dynamic spectrum conditions.

Despite these advancements, several gaps remain evident across the literature. A primary concern is the considerable variation in implementation strategies, performance metrics, and experimental settings across studies. This methodological heterogeneity undermines cross-comparison and weakens the external validity of reported outcomes. Moreover, many studies continue to emphasize algorithmic novelty at the expense of practical deployment considerations, such as scalability, real-time adaptability, and energy efficiency. Consequently, the operational maturity and deployment readiness of many proposed models remain limited, especially in edge computing and resource-constrained environments.

To strengthen the scientific rigor and practical impact of clustering research in mobile network optimization, future studies should prioritize the development of standardized benchmarking frameworks. In practice, such frameworks should incorporate shared open datasets, common performance indicators (e.g., spectral efficiency, latency, clustering stability, and energy consumption), and reproducible experimental configurations using containerized simulation environments (e.g., Docker, OMNeT++, or NS-3). Establishing such unified platforms would enable consistent cross-study evaluation and provide a foundation for quantitative and qualitative benchmarking of clustering algorithms under realistic network conditions.

Equally important is the need to balance competing performance objectives, notably accuracy, adaptability, and computational efficiency. Achieving this balance requires the integration of multi-objective optimization and context-aware clustering frameworks capable of dynamically adjusting to changing network conditions. Hybrid edge-cloud architectures, online learning mechanisms, and hierarchical clustering pipelines may offer viable pathways for maintaining high accuracy without compromising latency or energy efficiency.

Finally, as deep learning-based clustering methods continue to gain prominence, interpretability and transparency must be treated as first-class design objectives. Incorporating explainable AI (XAI) techniques, such as SHAP values, Layer-wise Relevance Propagation (LRP), and attention visualization, can significantly enhance model transparency by revealing how specific features (e.g., signal strength, interference, or mobility patterns) influence clustering outcomes. Furthermore, adopting prototype-based neural clustering and self-explainable architectures will improve user trust and facilitate integration into mission-critical, real-time network operations.

In conclusion, advancing clustering for mobile network optimization requires a shift from isolated, algorithm-centric research toward a systematic, standardized, and interpretable paradigm. By establishing common benchmarks, balancing performance trade-offs, and embedding explainability into model design, the research community can accelerate progress toward scalable, adaptive, and transparent clustering solutions for next-generation networks. Table 9 presents the identified gaps and future research recommendations in clustering for mobile network optimization.

Moreover, we conclude that the scalability limitations are particularly pronounced across several clustering paradigms. Density-based algorithms (e.g., DBSCAN, OPTICS) exhibit high computational complexity during neighborhood searches, making them impractical for large-scale, real-time datasets. Spectral clustering suffers from the cubic cost of eigen-decomposition, restricting its use to small or moderate datasets. Deep representation-based clustering, though effective in feature extraction, demands extensive computation and memory, challenging its deployment in edge or fog environments. In contrast, centroid-based methods like K-means are scalable but often fail to adapt to dynamic or high-dimensional network conditions. Addressing these challenges will require distributed, incremental, and edge-cloud cooperative clustering frameworks that maintain accuracy while supporting the scalability demands of next-generation mobile networks.

## Figures and Tables

**Table 1:** Summary of the main clustering algorithms.

Algorithm	Idea	Objective Function
DBSCAN	Clusters are defined as dense regions separated by low-density regions. No need to specify number of clusters. Outliers are naturally detected. Form clusters by density reachability.	*Nε*(*p*) = {*q∈D* ∣ ‖*p* − *q*‖ ≤ *ε*}, Where: - *p*: a data point (the “center” of interest) - *q*: any other data point in the dataset *D*. - ‖*p−q*‖: the distance between points *p* and *q* - (usually Euclidean distance, but can be any metric) - **ε**: a fixed distance threshold (epsilon) - *Nε*(*p*): the set of all points *q* in the dataset *D* that are within distance *ε* of *p*
K-Means	Partition the dataset into *k* clusters such that each point belongs to the cluster with the nearest centroid. Measures how compact the clusters are. K-Means minimizes this value to find the best partitioning	- The centroid: $\mu_{i}\;\;=\;\;\frac{1}{|C_{i}|}\sum_{x\in C_{i}}x$ - The objective: $J=\sum_{i=1}^{k}\sum_{x\in C_{i}}\|x-\mu_{i}{\|}^{2}$ Where: - *k*: number of clusters. - *C_i_*: set of data points assigned to cluster *i*. - *µ_i_*: centroid (mean) of cluster *i* - ‖*x*−*μ*_*i*_‖^2^: squared distance between a point *x* and its cluster centroid - The double summation: add up all squared distances of every point to its assigned centroid, across all clusters. - *J*: total sum of squared distances between data points and their cluster centroids
Bayesian Non-Parametric (DPMM)	Unlike K-Means (fixed *k*) or DBSCAN (density threshold ε), Bayesian non-parametric models (like Dirichlet Process Mixtures) let the number of clusters be determined by the data itself. Clusters are not pre-set.	Generative (stick/measure) view: $G∼DP(\alpha ,G_{0}),\theta_{n}|G∼G,x_{n}|\theta_{n}∼p(x|\theta_{n}).$ Assignment (CRP) view: $z_{n}∼CRP(\alpha ),\theta_{k}∼G_{0}(k=1,\ldots ,K),x_{n}|z_{n},\{\theta_{k}\}∼p(x|\theta_{z_{n}}).$ Posterior (complete form): $p(z,\theta |X)\propto p(X|z,\theta )\;p(\theta |G_{0})\;p(z|\alpha ),$ Where:
		$G_{0}$ = Prior over what a single cluster looks like (e.g., Gaussian mean/covariance)
		α = Controls number of clusters (small → few clusters, large → many)
		G = Random mixture of cluster components drawn from $DP(\alpha ,G_{0})$ CRP = Distribution over how data are assigned to clusters
		DPMM = Infinite mixture model where the number of clusters grows with the data
DEMC	Learn a nonlinear mapping that embeds data into a latent space where clustering structure is more separable. Incorporates manifold learning with deep networks.	*L* = *L*_rec_ + *λL*_cluster_ Where: - *L*_rec_: Reconstruction Loss - *L*_cluster_: Clustering Loss - *λ*: Trade-off parameter
Spectral clustering		Multi-way normalized cut objective: $$\underset{A_{1},\ldots ,A_{k}}{min}\sum_{i=1}^{k}\frac{cut(A_{i},{\bar{A}}_{i})}{vol(A_{i})},$$ where $cut(A,B)=\sum_{u\in A}\;\;\sum_{v\in B}\;\;w_{uv}$ and $vol(A)=\sum_{u\in A}\;\;d_{u}$. Relaxation → generalized eigenproblem (symmetric normalized Laplacian): $L_{sym}u=\lambda u,L_{sym}=I-D^{-1/2}WD^{-1/2},$ and the rows of the matrix formed by the first keigenvectors are clustered using k-means in the embedding space.

**Table 2:** SPICE Protocol Framework.

SPICE Element	Description
Setting (S)	Heterogeneous and dynamic mobile/5G network environments and beyond
Perspective (P)	Network engineers, researchers, and system designers.
Intervention (I)	Application of advanced clustering techniques (spectrum-based, density-based, deep representation-based).
Comparison (C)	Traditional clustering approaches (e.g., K-means, PSO) or baseline methods
Evaluation (E)	Improvements in feature discovery, anomaly detection (e.g., accuracy, precision, recall), and adaptive Quality-of-Service (QoS) metrics (e.g., throughput, latency, handover success rate, resource utilization efficiency).

**Table 3:** PRISMA stages explained.

PRISMA Stage	Figure Element/Description	Count (n)
Identification	Records identified, duplicates removed	10,238 → 9738
Screening	Title/abstract screened, excluded	9738 → 9238
Eligibility	Full text assessed, excluded with reasons	500 → 460
Inclusion	Final included studies (qualitative synthesis)	40

**Table 4:** Exclusion reasons and counts.

Exclusion Reason	Count (n)
Not focused on clustering in mobile networks	210
No empirical/quantitative evaluation	120
Outside date range/out of scope	85
Non-peer-reviewed/not accessible	30
Duplicate/data error	15
Total excluded	460

**Table 5:** AMSTAR quality appraisal summary.

AMSTAR Question	Yes/No	Comments
Was an ‘a prior’ design provided	Yes	Screening and extraction criteria were stated upfront
Was there duplicate study chosen and data extraction?	No	No explicit mention of duplicate/independent screening
Was a complete literature search implemented?	Yes	Searched semantic Schoolar through 126 million papers
Was the status of the publication used as an inclusion criterion?	No	No restriction based on the publication status mentioned.
Was a list of studies (included and excluded) provided?	No	Only characteristics of included studies were summarized.
Were the characteristics of the included studies provided?	Yes	Table of computational method, domain, etc. included.
Was the scientific quality of the studies included assessed?	Yes	Bias and resource requirement evaluations mentioned.
Was the scientific quality used appropriately in formulating conclusions?	Yes	Conclusions carefully considered study quality and limitations.
Were the methods used to combine findings appropriate?	Yes	Grouped by computational method, domain, and performance.
Was the likelihood of publication bias assessed?	No	No formal assessment of publication bias performed.
Was conflict of interest included?	No	No conflicts of interest were mentioned in the report.

To increase rigor, we also used PRISMA for reporting transparency (protocol registration on OSF.

**Table 6:** Characteristics of the Included Studies.

Study Reference	Study	Network Environment	Clustering Technique	Application Focus	Key Findings	Full Text Retrieved
[15]	Ali et al., 2018	Multi-channel cognitive radio networks (MCRNs)	Spectral clustering (Bayesian non-parametric)	Quality of Service (QoS) level identification	Effective in identifying QoS levels supported over available licensed channels	No
[16]	Gajic et al., 2015	Mobile networks	Incremental time-aware clustering	Anomaly detection	Improved → detection of different types of anomalies in cell functionality	No
[17]	Sivavakeesar and Pavlou, 2004	Multihop mobile ad hoc networks	Prediction-based clustering	QoS support	Proposed (p, t, → d)-clustering model for consistent network view	No
[18]	Nivitha et al., 2020	Cellular networks	Dynamic clustering in Federated	Handover prediction	Improved forecasting performance by 3%	Yes
[19]	Fernández Maimó et al., 2018b	5G mobile networks	Deep learning-based	Anomaly detection	Self-adaptive system for real-time anomaly detection	No
[20]	Kaleibar and St-Hilaire, 2024	Vehicular Cloud Networks (VCNs)	Adaptive clustering	Dynamic service provisioning	Achieved more stable clusters and lower overhead	No
[21]	Fernández Maimó et al., 2018a	5G	Deep learning-based	Anomaly detection	High precision and recall for known botnets, reasonable generalization for unknown botnets	Yes
[22]	Sun et al., 2020	Internet of Spectrum Devices (IoSD)	Spectral clustering (K-means and hierarchical)	Spectrum prediction	Improved → inference performance on accuracy and runtime overhead	No
[23]	Yin et al., 2020	Mobile edge computing	Hybrid (denoising auto-encoder with fuzzy clustering)	QoS prediction	Improved performance and reduced overfitting problem	No
[24]	Caleb and Thangaraj, 2023	Future ultra-dense mobile networks	No mention found	Quality of Experience (QoE)-motivated anomaly detection	Proposed a user-centric approach for anomaly detection	No
[25]	Elsayed and Erol-Kantarci, 2020	5G mmWare	Density-based (DBSCAN)	Resource allocation	Improved latency, reliability, and rate for URLLC and eMBB users	Yes
[26]	Ren and Xu, 2019	5G ultra-dense networks	Density-based (DBSCAN) and PSO	Clustering for CoMP	Achieved → higher system throughput compared to modified K-means scheme	No
[27]	Kim et al., 2021	Internet of Things (IoT)	Hybrid (clustering and reinforcement learning)	Anomaly detection	Proposedframework for automated learning of anomaly detection	No
[28]	Kassan et al., 2023	LTE networks	Hybrid (co-clustering and logistic regression)	Anomaly forecasting	Compared performance with LSTM and TCN approaches	No
[29]	Benslimane et al., 2011	Integrated VANET-UMTS	Hybrid (direction, RSS, distance)	Gateway management	Improved data packet delivery ratios, throughput, and reduced delay	Yes
[30]	Xu et al., 2017	Wireless Sensor Networks (WSNs) and IoT in 5G	Survey of various techniques	Energy efficiency, QoS, and QoE	Identified challenges in applying clustering to IoT in 5G environments	No
[31]	Stenhammar et al., 2024	Cellular networks	Geographical segment clustering with Federated Learning	Predictive QoS for connected vehicles	Outperformed common predictive approach with a single global model	No
[32]	Padmanabhan et al., 2016	Vehicular Ad-hoc Network (VANET)	Dynamic multi-clustering	QoS improvement	Improved packet delay, throughput, and packet loss ratio	
[33]	Ali et al., 2024	Mobile Ad Hoc Networks (MANETs)	Deep Representation based clustering	Adaptive clustering for data collection	Improved delivery rate (up to 89.4%) and reduced packet drop rates (>70%)	Yes
[34]	Aljadhai and Znati, 2001	Wireless (picoand micro-cellular)	No mention found	QoS provisioning	Integrated mobility model with service model for efficient resource utilization	No
[35]	Almobaideen et al., 2011	Mobile Ad Hoc Networks (MANET)	No mention found	QoS support	Improved overall network throughput and decreased end-to-end delay	No
[36]	Aziz and Bestak, 2024	5G	Spectrum clustering (K-means)	Anomaly detection and prediction	Achieved 96% No accuracy in anomaly detection using CDR data	No
[37]	Balakrishnan et al., 2021	Ad-hoc networks	Deep representation-based clustering (Deep Ensemble Model for Clustering, DEMC)	Routing misbehavior detection	Proposed DEMC for better anomaly detection in resource-constrained environments	No
[38]	Casas et al., 2016	Cellular networks	K-means clustering	Mobile apps anomaly detection	Achieved ~70% detection rate without false alarms, ~85%	Yes
[39]	Cretu-Ciocarlie et al., 2013	Cellular networks	Ensemble method	Cell anomaly detection	Improved detection quality over univariate and multivariate methods	No
[40]	Hussain et al., 2019	5G	Deep learning-based	Anomaly detection	Achieved 98.8% accuracy with 0.44% false positive rate	No
[41]	Kajó et al., 2021	Mobile networks	representation-based (Deep Attentive Neural Clustering of Embeddings, DANCE)	Clustering mobile network data	Outperformed state-of-the-art deep clustering algorithms	No
[42]	Kajó et al., 2022	Mobile networks	Deep representation-based (DANCE)	Clustering mobile network data	Improved performance in mobile user behavior clustering task	No
[43]	Moulay et al., 2020	Commercial mobile networks	Learning K-means clustering	Networking anomaly detection	Achieved 85% accuracy in decision tree for anomaly identification	Yes
[44]	Moysen et al., 2016	4G and 5G	Ensemble regression	QoS prediction	Proposed approach for improving QoS-based network planning	No
[45]	Moysen et al., 2020	LTE (4G)	Unsupervised learning	Mobility-related anomaly detection	Effective in identifying cells With mobility-related performance degradation	No
[46]	Murudkar and Gitlin, 2019a	5G and beyond, Self-Organizing Networks (SONs)	No mention found	QoE prediction and anomaly detection	Achieved accuracy score greater than 99%	No
[47]	Murudkar and Gitlin, 2019b	LTE (4G), potentially 5G	No mention found	QoE-driven anomaly detection	Proposed a user-centric approach for anomaly detection	No
[48]	Oldmeadow et al., 2004	No mention found	Adaptive clustering	Network intrusion detection	Developed time-varying modification of standard clustering technique	No

**Table 7:** Clustering techniques and their applications.

Technique Type	Implementation Approach	Performance Metrics	Adaptation Capability
Spectral clustering	Bayesian non-parametric, K-means	QoS level identification accuracy, Anomaly detection accuracy (96%)	Adaptive to channel conditions
Density-based Clustering	DBSCAN	Latency improvement, Reliability (PLR), Data rate	Online clustering for dynamic environments
Deep Representation-based Clustering	Deep Ensemble Model for Clustering (DEMC), Deep Attentive Neural Clustering of Embeddings (DANCE), Various Deep Learning architectures	Anomaly detection accuracy, Clustering performance improvement	Self-adaptive to traffic fluctuations
Hybrid Approaches	Clustering + Reinforcement Learning, Co-clustering + Logistic Regression	Accuracy, False Positive Rate	Adaptive to changing network conditions
K-means Clustering	Standard K-means	Detection rate, Accuracy	Limited adaptation, often combined with other techniques
Ensemble Methods	Multiple learners, Ensemble regression	Improved detection quality	Adaptive through ensemble diversity
Federated Learning-based	Clustered Federated Learning, Dynamic clustering in Federated Learning	Prediction accuracy improvement (43%)	Adaptive to local data characteristics
Time-aware Clustering	Incremental approach	Anomaly detection performance	Adaptive to temporal network changes
Prediction-based Clustering	Mobility prediction	QoS support metrics	Adaptive to node mobility patterns

**Table 8:** Network performance enhancement.

Enhancement Area	Implementation Method	Observed Benefits	Limitations
QoS Prediction	Ensemble regression, Deep Learning, Federated Learning	Better PRB/MB prediction, Generalization	Lack of standard QoS metrics
Anomaly Detection	K-means, Deep Learning, Hybrid Approaches	High accuracy (98.8%), Low false positive rates	Real-time detection challenges
Resource Allocation	DBSCAN & LSTM-DRL, Adaptive clustering	Improved latency and reliability, more stable clusters	Complexity in handling heterogeneous network resources
Energy Efficiency	Survey of various techniques	Potential for improved network longevity	Trade-off between energy efficiency and other performance metrics
Handover Prediction	Dynamic clustering in Federated Learning	43% improvement in prediction accuracy	Privacy concerns in distributed learning environments
Spectrum Management	Bayesian non-parametric clustering, K-means and hierarchical clustering	Effective QoS level Identification, Improved spectrum prediction	Challenges in real-time adaptation to spectrum dynamics
Network Security	NetWalk, Ensemble Methods	Real-time anomaly detection, Improved detection quality	Balancing detection accuracy with false positive rates
QoE Optimization	QoE-driven clustering	User-centric optimization, High accuracy in QoE prediction	Complexity in quantifying and predicting subjective QoE metrics
Mobility Management	Prediction-based clustering	Improved QoS support in mobile environments	Challenges in accurate mobility prediction in complex scenarios
Traffic Prediction	ARIMA + Clustering	Improved prediction accuracy with anomaly-free data	Sensitivity to anomalies

**Table 9:** Identified gaps and future research recommendations in clustering for mobile network optimization.

Identified Gaps	Recommendations
High variation in implementation strategies, performance metrics, and experimental settings	Develop standardized benchmarking frameworks, including common datasets and unified evaluation protocols, to ensure comparability and reproducibility
Limited focus on scalability, real-time processing, and energy efficiency	Design clustering models optimized for real-time performance, scalability, and energy efficiency, especially in edge and resource-constrained environments
Predominant use of traditional clustering methods without significant methodological innovation	Encourage research into adaptive, hybrid, and deep representation-based clustering methods suited for complex and dynamic mobile network conditions
Poor generalizability and external validity due to context-specific evaluations	Validate models across diverse network settings (e.g., IoT, vehicular, and 5G environments) to improve external validity and generalizability
Lack of interpretability and transparency in deep learning-based clustering approaches	Prioritize the development of explainable and interpretable clustering models to support trust and usability in critical real-time applications
Insufficient empirical testing in real-world scenarios	Increase the use of real-world deployments or realistic testbeds to assess the practical effectiveness of proposed clustering techniques

## Data Availability

The datasets presented in this article are not readily available because the data are part of an ongoing study. Requests to access the datasets should be directed to claudenwj@gmail.com.
